# Supplementary material for: Discovery, Genomic Sequence Characterization and Phylogenetic Analysis of Novel RNA Viruses in the Turfgrass Pathogenic Colletotrichum spp. in Japan
Source: Viruses. 2022 Nov 20;14(11):2572. doi: 10.3390/v14112572 (PMC9698584; doi:10.3390/v14112572)
Supplement: Supplementary file 1 [file viruses-14-02572-s001.zip › viruses-1953051-Table S1-S10.pdf]

Supplementary Table S1: Primers used in RT-PCR validation of mycovirus genome segments identified in FLDS

**Contigs**	**Length (nt)**	**Target**	**FP**	**RP**	**Tm**
S 264_5_full	891	363-1234	TGCGAACAAACGTTTCCGAC	TCGAAGAACTTCGCTGCCTT	59.97/59.97
S264_1_full	831	211-1042	GAGACTGATTTCGCTGGCCT	GCGCTACCACCTTCGTACTT	60/60
S264_2_full	619	207-825	CCTGGTCGCTTTTCTCCAGT	GATCATAGTGTCGGCGTCGT	60/60
S264_3_full	711	780-1471	TCAGAGCAGTCTTTGGCGTT	AGCTGAGTACTGACATGCCG	60/60
S264_4_full	717	549-1265	ACGTTGGATTTCCCCGATGT	GCCATGCTGATGCACATCTC	60/60
S264_7_full	1803	360-1191	AACGTCTTAAACGCCTCCGT	TTCACGGGTACCAAGTCTGC	60/60
S264_6_full	1755	488-1363	ACGGACCGAGACTGTCACTA	CTCCAGGACGCTGTTTGTCT	60/60
S264_8_full	797	4478-5275	TGGACAGACGAACAGCACAATC	CATGACCGAGAACAAACGGC	58/57
S288_2_full	738	3187-3924	CAGCGCATATGTCCTGCAAC	TGCCAGATTGCATGCCCTTA	60/60
S288_1_full	2947	1975-2585	GACCGCAAAGGCATTCCAAG	AGCTAGGAGCGGTGGCAATC	57/59

Supplementary Table S2: Mapping report of FLDS of dsRNA of MBCT-264 and MBCT-288

	**Count**	**Percentage of reads**	**Average length**	**Number of bases**
**dsRNA of MBCT-264**				
Total reads	1,536,110	100.00%	125.97	93,496,749
Virus contigs	8	-	2,362.12	18,897
Mapped reads to virus contigs	1,523,226	99.16%	126.23	192,279,571
Not mapped reads to virus contigs	12,884	0.84%	94.47	1,217,178
Reads in pairs	760,412	49.50%	376.81	96,159,304
Broken paired reads	762,814	49.66%	126.01	96,120,267
**dsRNA of MBCT-288**				
Total reads	1,445,468	100.00%	121.60	175,765,255
Virus contigs	2	-	5,075.00	10,150
Mapped reads to virus contigs	1,312,155	90.78%	121.91	159,959,167
Not mapped reads to virus contigs	133,313	9.22%	118.56	15,806,088
Reads in pairs	732,714	50.69%	370.01	89,364,439
Broken paired reads	579,441	40.09%	121.83	70,594,728

Supplementary Table S3: Virus contigs identified from FLDS-reads generated from *Colletotrichum* strains MBCT-264 and MBCT-288 dsRNAs

SL	Contig	Length (bp)	Counts	5’	3’
FLDS of dsRNA of MBCT-264					
CaPV1					
1	S264_5_full	1728	171656	CGTTTTGAAGGGCAAAAACG	CTCGTTCAACACCAAGAACC
2	S264_1_full	1388	753283	CGTTTAGTTAAGTGGACTCA	TTCTGTTCAACAAGAACTCC
3	S264_2_full	1160	338478	CGTTTAGAGACGGTGTTCAT	TGTCTTTCAACAAATAAACC
CaPV2					
4	S264_3_full	1958	116041	AGAATTTCTTTCCTAATACT	AAAAAACGAAAAAAAAATAAA
5	S264_4_full	1872	99372	AGAATTTCTTTCCTATTTAA	AAAAAAAAGGAAAAAAAAACATAAA
6	S264_7_full	1803	15810	AGAATTTCTTCTCCTAGATA	AAAAAAAAGTAAAAAAATAAA
7	S264_6_full	1755	19427	AGAATTTCTTCTCCTAAAAT	AAATGAAAAAAAAAAAATAAA
CaNSRV1					
8	S264_8_full	7233	8753	CCCTTGGCTAATTACACGAAA	TTGCCAAGGGGGTCTTTGTG
FLDS of dsRNA of MBCT-288					
CaNSRV2					
9	S288_2_full	7203	247752	ATAAAAGAAAAGAAGACCTT	TTTATACAACTTATTTCTAA
10	S288_1_full	2947	1064402	ACGAAAAAGAAAGAAACCCT	AAAAAAATCAATCTTATTTCTAA

Supplementary Table S4: BLASTp search of ORF1(RdRp) of Colletotrichum associated partitivirus 1 (CaPV1) using the non-redundant protein sequences database of NCBI

Virus name	Query Cover	E value	Per. ident	Acc. Len	Accession
Colletotrichum gloeosporioides partitivirus 1 (CgPV1)	100%	0	88.95	525	QED88095.1
Plasmopara viticola lesion associated Partitivirus 4 (PvLaPV4)	98%	0	72.78	523	QHD64807.1
Plasmopara viticola lesion associated Partitivirus 3 (PvLaPV3)	98%	0	73.35	523	QHD64801.1
Erysiphe necator associated partitivirus 7 (EnaPV7)	98%	0	72.96	523	QJW70316.1
Macrophomina phaseolina partitivirus 1 (MpPV1)	96%	0	71.94	512	QKO02079.1
Ustilaginoidea virens partitivirus 3 (UvPV3)	96%	0	66.53	522	AGJ03719.1
Ustilaginoidea virens partitivirus 2 (UvPV2)	96%	0	64.62	529	YP_008327312.1
Aspergillus flavus partitivirus 1 (AfPV1)	96%	0	53.31	548	QDE53634.1
Botryosphaeria dothidea virus 1 (BdV1)	98%	0	52.48	544	AIE47694.1
Colletotrichum acutatum RNA virus 1 (CaRV1)	96%	0	52.22	540	AGL42312.1
Aspergillus niger partitivirus 1 (AnPV1)	96%	0	51.84	542	BDF97658.1
Fusarium cerealis partitivirus 1 (FcPV1)	97%	9.00E-177	49.81	528	QOL02536.1
Erysiphe necator associated gammapartitivirus 1 (EnaGPV1)	87%	9.00E-71	36.62	543	QKK35392.1
Aspergillus ochraceous virus (AoV)	86%	6.00E-68	34.42	539	AYP71818.1
Aspergillus ochraceous virus (AoV)	86%	1.00E-67	34.2	539	YP_009665972.1
Aspergillus ochraceous virus (AoV)	86%	1.00E-67	33.98	539	ABC86749.1
Colletotrichum truncatum partitivirus 1 (CtPV1)	85%	3.00E-67	35.87	539	ALF46547.1
Phyllosticta citriasiana partitivirus 1 (PcPV1)	85%	2.00E-65	35.64	535	AZT88588.1

Fusarium mangiferae partitivirus 2 (FmPV2)	85%	3.00E-65	34.57	539	UBZ25878.1
Sodiomyces alkalinus partitivirus 2 (SaPV2)	85%	7.00E-65	34.93	520	ATP85067.1
Botryotinia fuckeliana partitivirus 1 (BfPV1)	85%	9.00E-65	34.35	540	QJQ28886.1
Laodelphax striatella associated partitivirus (LsaPV)	86%	2.00E-64	32.9	538	AZU96334.1
Aspergillus fumigatus partitivirus 1 (AfuPV1)	86%	1.00E-59	32.19	542	CAY25801.2
Ophiostoma partitivirus 1 (OpPV1)	87%	1.00E-59	31.71	539	YP_009508238.1
Gremmeniella abietina RNA virus MS1 (GaRVMS1)	84%	2.00E-63	33.56	539	NP_659027.1

Supplementary Table S5: BLASTp search of ORF2 (Putative hypothetical /capsid protein) of Colletotrichum associated partitivirus 1 (CaPV1**)** using the non- redundant protein sequences database of NCBI

Virus Name	Query Cover	E value	Per. Ident	Acc. Len	Accession
Colletotrichum gloeosporioides partitivirus 1 (CgPV1)	100%	0	73.4	375	QED88096.1
Plasmopara viticola lesion associated Partitivirus 4 (PvLaPV4)	100%	2.00E-172	65.33	374	QHD64811.1
Phoma matteuciicola partitivirus 1 (PmPV1)	100%	1.00E-166	63.4	376	QDK65070.1
Erysiphe necator associated partitivirus 7 (EnaPV7)	98%	5.00E-155	59.95	373	QJW70323.1
Ustilaginoidea virens partitivirus 2 (UvPV2)	99%	3.00E-142	56.65	375	YP_008327313.1
Ustilaginoidea virens partitivirus (UvPV)	99%	1.00E-140	56.65	375	AGO04404.1
Macrophomina phaseolina partitivirus 1 (MpPV1)	99%	8.00E-133	53.23	374	QKO02080.1
Ustilaginoidea virens mycovirus (UvMV)	97%	2.00E-112	47.96	371	AGJ03720.1
Aspergillus niger partitivirus 1 (AnPV1)	97%	1.00E-25	27.22	371	BDF97659.1
Colletotrichum acutatum RNA virus 1 (CaRV1)	97%	3.00E-21	25.41	370	AGL42313.1
Botryosphaeria dothidea virus 1 (BdV1)	97%	1.00E-18	26.4	370	AIE47695.1
Aspergillus flavus partitivirus 1 (AfPV1)	97%	6.00E-16	23.99	369	QDE53635.1
Fusarium cerealis partitivirus 1 (FcPV1)	97%	2.00E-10	21.93	378	QOL02537.1

Supplementary Table S6: BLASTp searches of the deduced amino acid sequences of RdRp of Colletotrichum associated partitivirus 2 (CaPV2) and related partitiviruses in nr database of protein sequences

Scientific Name	Query Cover	E value	Per. ident	Acc. Len	Accession
Plasmopara viticola lesion associated Partitivirus 9 (PvaPV9)	100%	0	90.62	608	QHD64790.1
Fusarium solani partitivirus 2 (FsPV2)	99%	0	87.64	608	BAQ36631.1
Monilinia partitivirus A (MoPVA)	99%	0	61.9	604	QED42950.1
Hygrophorus penarioides partitivirus 1 (HpPV1)	95%	0	56.72	607	QQO86653.1
Partitiviridae sp.	99%	0	54.92	611	UDL14345.1
Rhizoctonia solani partitivirus 7 (RsPV7)	99%	0	54.95	609	QDW81313.1
Sarcosphaera coronaria partitivirus (ScPV)	95%	0	55.02	634	QLC36809.1
Rosellinia necatrix partitivirus 7 (RnPV7)	99%	0	52.88	608	BAT32942.1
Rhizoctonia oryzae-sativae partitivirus 5 (RoPV	99%	0	51.96	613	BCY26960.1
Rosellinia necatrix partitivirus 22 (RnPV22)	95%	0	52.84	613	BBU59852.1
Sarcosphaera coronaria partitivirus (ScPV)	99%	0	48.78	618	QLC36810.1
Rhizoctonia solani partitivirus 1 (RsPV1)	93%	2.00E-179	49.74	603	AND83003.1
Rhizoctonia solani dsRNA virus 18 (RsDRV18)	94%	5.00E-176	47.1	630	UIW13809.1
Sclerotinia sclerotiorum partitivirus S (SsPVs)	91%	5.00E-163	47.24	580	YP_003082248.1
Flammulina velutipes isometric virus (FvIV)	95%	7.00E-159	45.39	587	BAH08700.1
Rosellinia necatrix partitivirus 26 (RnPV26)	99%	1.00E-155	43.59	584	QMU26430.1

Scutellospora partitivirus B (SPVB)	92%	2.00E-147	45.07	531	QED43041.1
Ceratobasidium partitivirus (CbPV)	89%	3.00E-144	44.28	548	AOX47573.1
Erysiphe necator partitivirus 2 (EnPV2)	92%	2.00E-139	42.15	577	ATS94403.1
Botrytis cinerea partitivirus 2 (BcPV2)	91%	1.00E-122	43.14	579	QBA69894.1

Supplementary Table S7: BLASTp search of ORF2 (Coat /capsid protein) of Colletotrichum associated partitivirus 2 (CaPV2) using the non-redundant (nr) protein sequences database of NCBI

Scientific Name	Query Cover	E value	Per. ident	Acc. Len	Accession
Plasmopara viticola lesion associated Partitivirus 9 (PvLaPV9)	99%	0	77.74	541	QHD64805.1
Plasmopara viticola lesion associated Partitivirus 2 (PvLaPV2)	89%	0	69.48	485	QHD64794.1
Lichen partiti-like RNA virus 1 (LplRV1)	97%	8.00E-96	35.79	534	BCD56382.1
Rosellinia necatrix partitivirus 5 (RnPV5)	93%	3.00E-33	26.22	576	BCB16796.1
Hygrophorus penarioides partitivirus 1 (HpPV1)	84%	9.00E-28	25.5	574	QQO86654.1
Rosellinia necatrix partitivirus 22 (RnPV22)	87%	4.00E-27	24.33	584	BBU59853.1
Sarcosphaera coronaria partitivirus (ScPV)	93%	5.00E-27	24.55	585	QLC36765.1
Rhizoctonia oryzae-sativae partitivirus 5 (RosPV5)	82%	2.00E-24	23.81	567	BCY26961.1
Trichoderma atroviride partitivirus 1 (TaPV1)	90%	6.00E-20	22.59	577	AYQ58322.1
Caloscypha fulgens partitivirus 7 (CfPV7)	56%	1.00E-19	25.08	569	QOI17260.1
Rhizoctonia solani partitivirus 7 (RsPV7)	68%	4.00E-17	23.04	567	QDW81314.1
Pear alphapartitivirus (PAPV)	53%	3.00E-15	28.91	491	BBA66578.1
Aspergillus flavus partitivirus 2 (AfPV2)	70%	1.00E-12	21.65	509	UAW09572.1
Medicago sativa alphapartitivirus 1 (MsAPV1)	52%	2.00E-12	26.96	491	ATJ00051.1
Rosellinia necatrix partitivirus 23 (RnPV 23)	66%	4.00E-12	22.68	514	BBU59855.1
Soybean leaf-associated partitivirus 2 (SLaPV2)	54%	7.00E-11	25.25	496	ALM62248.1
Rhizoctonia oryzae-sativae partitivirus 1 (RosPV1)	40%	4.00E-10	28.57	485	AYV61426.1
Rhizoctonia oryzae-sativae partitivirus 1 (RosPV1)	47%	1.00E-09	26.87	485	BCY26953.1
Rosellinia necatrix partitivirus 12 (RnPV12)	60%	6.00E-09	25.36	533	BBU59834.1
Heterobasidion partitivirus 21 (HbPV21)	30%	7.00E-09	30.3	495	UHK02581.1
Rosellinia necatrix partitivirus 24 (RnPV24)	42%	9.00E-09	23.29	498	BBU59857.1

Supplementary Table S8. BLASTp analysis of Colletotrichum associated negative stranded RNA virus 1 (CaNSRV-1) RdRp in the NCBI nr protein database

Scientific Name	Query Cover	E value	Per. ident	Acc. Len	Accession
Grapevine associated cogu-like virus 2(GaCLV2)	99%	0	69.78	2391	QIJ25707.1
Grapevine associated cogu-like virus 3 (GaCLV3)	99%	0	69.73	2385	QIJ25710.1
Laurel Lake virus (LLV)	99%	0	61.82	2384	YP_009667028.1
Grapevine associated cogu-like virus 4 (GaCLV4)	94%	0	64.36	2235	QMP81963.1
Hainan phenui-like virus 7 (HPLV7)	99%	0	61.29	2404	QYF49558.1
Watermelon crinkle leaf-associated virus 1 (WCLaV1)	81%	0	35.31	2182	ASY01340.1
Citrus concave gum-associated virus (CCGaV)	89%	0	33.47	2184	QDK54399.1
Citrus leaf flecking-associated virus (CLFaV)	89%	0	33.46	2184	QBZ68778.1
Citrus virus A (CVA)	89%	0	32.24	2184	QPF47541.1
Watermelon crinkle leaf-associated virus 2 (WCLaV2)	77%	0	34.29	2198	ASY01343.1
Yunnan Paris negative-stranded virus (YPNSV)	82%	0	34.32	2181	QVU28732.1
Brassica campestris chinensis coguvirus 1 (BCCCV1)	77%	0	34.58	2200	UFE16634.1
Sanya phenuivirus 1 (SPV1)	83%	0	32.55	2206	UHM27566.1
Grapevine associated cogu-like virus 1 (GaCLV1)	73%	0	34.15	2197	QIJ25704.1
Botrytis cinerea bocivirus 1 (BcBV1)	77%	0	32.32	2211	QJT73693.1
Lentinula edodes negative-strand RNA virus 3 (LeNSRV3)	79%	0	31.49	2308	QOX06031.1
Alternaria tenuissima negative-stranded RNA virus 2 (AtNSRV2)	74%	3.00E-117	24.31	2063	QDB75016.1
Bhanja virus (BhV)	68%	1.00E-99	24.59	2082	AGC60111.1

Supplementary Table S9:. BLASTp analysis of RdRp of Colletotrichum associated negative stranded RNA virus 2 (CaNSRV-2) RdRp in the NCBI nr protein database

Virus Name	Query Cover	E value	Per. Ident	Acc. Len	Accession
Fusarium poae negative-stranded virus 1 (FpNSV1)	100%	0	65.69	2346	YP_009272911.1
Plasmopara viticola lesion associated mycoophiovirus 5 (PvLaMoV5)	100%	0	65.64	2346	QJX19791.1
Cladosporium cladosporioides negative-stranded RNA virus 1(CcNSRV1)	100%	0	65.26	2359	QDB75017.1
Grapevine-associated negative single-stranded RNA virus 4 (GaNSRV4)	93%	0	65.89	2203	QXN75414.1
Plasmopara viticola lesion associated mycoophiovirus 4 (PvLaMoV4)	100%	0	59.82	2346	QJX19790.1
Erysiphe necator associated negative-stranded RNA virus 12 (EnaNSRV12)	100%	0	59.69	2346	QJW70351.1
Sclerotinia sclerotiorum ophiovirus like virus 1 (SsOLV1)	98%	0	42.78	2365	QUE49149.1
Sclerotinia sclerotiorum negative-stranded RNA virus 9 (SsNSRV9)	98%	0	42.76	2365	AYA73395.1
Sanya aspivirus 1 (SAV1)	90%	0	31.01	2664	UHM27585.1
Plasmopara viticola lesion associated mycoophiovirus 1 (PvLaMoV1)	92%	0	29.33	2739	QJX19787.1
Rhizoctonia solani negative-stranded virus 2 (RsNSV2)	79%	0	31.69	2381	ALD89130.1
Rhizoctonia solani negative-stranded virus 3 (RsNSV3)	81%	0	30.8	2380	ALD89111.1
Rhizoctonia solani negative-stranded virus 1 (RsNSV1)	91%	0	29.14	2411	ALD89129.2

Phlebiopsis gigantea negative-strand virus 1 (PgNSV1)	82%	0	30.21	2375	UJT31808.1
Culex tritaeniorhynchus Aspiviridae-related virus (CtArV)	74%	0	32.59	2388	BBQ04833.1
Grapevine-associated serpento-like virus 1 (GaSLV 1)	92%	0	28.05	2377	QXN75438.1
Soybean thrips negative-stranded RNA virus 7 (StNSRV7)	78%	1.00E-156	26.49	2941	QQO81450.1
Soybean thrips negative-stranded RNA virus 1 (StNSRV1)	78%	2.00E-153	26.32	2942	QQX28961.1
Soybean thrips negative-stranded RNA virus 3 (StNSRV3)	62%	5.00E-150	28.12	2942	QQX28974.1
Wilkie ophio-like virus 1 (WOpV1)	79%	3.00E-149	26.32	2061	ASA47457.1
Mirafiori lettuce big-vein ophiovirus (MiLBVOV)	43%	2.00E-57	24.38	2280	QZA75361.1
Lettuce ring necrosis virus (LRNV)	43%	9.00E-54	24.06	2277	QTP72396.1
Ranunculus white mottle virus (RWMV)	41%	5.00E-47	22.76	2291	AWJ64314.1
Blueberry mosaic associated virus (BMaV)	19%	5.00E-42	29.25	2337	BAV13384.1
Citrus psorosis ophiovirus (CPOV)	48%	3.00E-41	22.11	2416	AXJ14438.1
Soybean cyst nematode nyami-like virus (SCNNLV)	21%	8.00E-13	22.42	2064	AVK42875.1
Chimay rhabdovirus (ChRV)	22%	1.00E-07	23.24	2199	AVM86063.1
Wenling chuvirus-like virus 2 (WCLV2)	19%	1.00E-04	20.69	2212	YP_009333151.1
Tacheng Tick Virus 6 (TTV6)	13%	0.002	25.42	2083	YP_009304420.1

Supplementary Table S10: BLASTp analysis of ORF7 of CaNSRV-2

Description	Scientific Name	Query Cover	E value	Per. ident	Acc. Len	Accession
hypothetical protein E4U09_005733 [Claviceps aff. purpurea]	Claviceps aff. purpurea	92%	3.00E-116	54.58	308	KAG6288140.1
hypothetical protein E4U34_002436 [Claviceps purpurea]	Claviceps purpurea	92%	4.00E-114	54.25	308	KAG6221060.1
hypothetical protein E4U12_002732 [Claviceps purpurea]	Claviceps purpurea	92%	5.00E-114	53.92	308	KAG6133501.1
hypothetical protein E4U57_006530 [Claviceps arundinis]	Claviceps arundinis	92%	7.00E-114	55.56	306	KAG5963027.1
hypothetical protein E4U11_003042 [Claviceps purpurea]	Claviceps purpurea	92%	3.00E-113	53.92	308	KAG6161930.1
hypothetical protein E4U36_005246 [Claviceps purpurea]	Claviceps purpurea	92%	9.00E-113	53.92	308	KAG6179915.1
hypothetical protein E4U22_003398 [Claviceps purpurea]	Claviceps purpurea	92%	1.00E-112	52.94	308	KAG6310255.1
hypothetical protein E4U40_001024 [Claviceps sp. LM458 group G5]	Claviceps sp. LM458 group G5	89%	3.00E-112	54.55	303	KAG6036368.1
hypothetical protein E4U30_005859 [Claviceps sp. LM220 group G6]	Claviceps sp. LM220 group G6	92%	4.00E-112	52.61	308	KAG6099838.1
hypothetical protein E4U24_002570 [Claviceps purpurea]	Claviceps purpurea	92%	4.00E-112	53.59	308	KAG6248664.1
hypothetical protein [Toensbergia leucococca]	Toensbergia leucococca	61%	4.00E-52	45.54	210	MCJ1231429.1
hypothetical protein EPUL_006619 [Erysiphe pulchra]	Erysiphe pulchra	88%	6.00E-42	30.98	423	POS82076.1
