# Supplementary figures and images for: Discovery, Genomic Sequence Characterization and Phylogenetic Analysis of Novel RNA Viruses in the Turfgrass Pathogenic Colletotrichum spp. in Japan
Source: Viruses. 2022 Nov 20;14(11):2572. doi: 10.3390/v14112572 (PMC9698584; doi:10.3390/v14112572)

### CaPV1

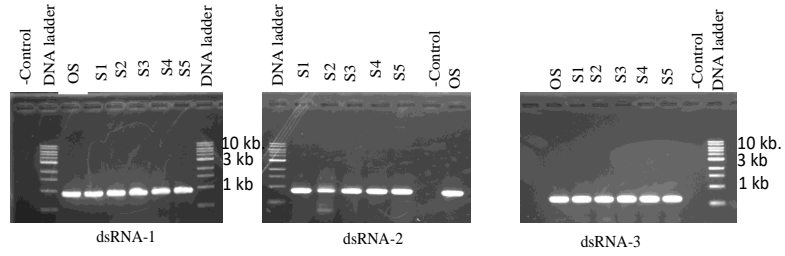

### CaPV2

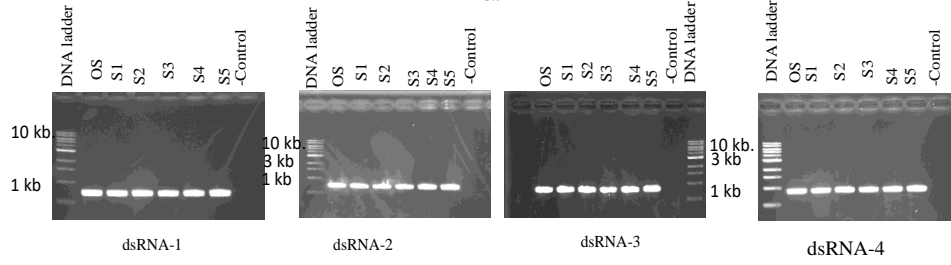

### CaNSRV1

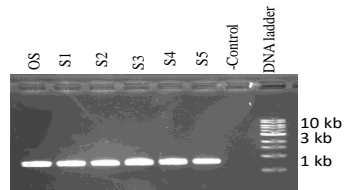

### CaNSRV2

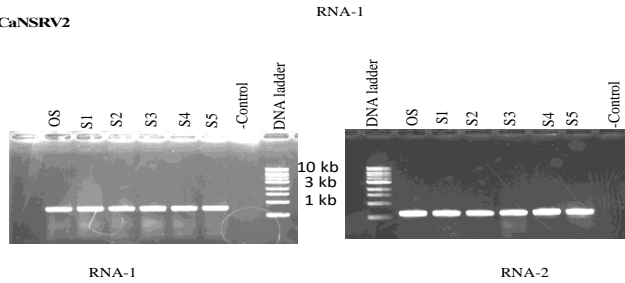

Supplement: Supplementary file 1 [file viruses-14-02572-s001.zip › viruses-1953051-Figure S1.pdf]
